# A voyage in the Far East

**DOI:** 10.1192/j.eurpsy.2022.1630

**Published:** 2022-09-01

**Authors:** G. Rodríguez Menéndez, C. García Bernal, M. Sevilla Fernández

**Affiliations:** 1 Hospital Universitario Virgen Macarena, Psychiatry, Sevilla, Spain; 2 Hospital Universitario Virgen del Rocío, Psychiatry, Sevilla, Spain

**Keywords:** Sevilla, Psychosis, immigration, journey

## Abstract

**Introduction:**

Patient who comes to the Hospital accompanied by the Emergency Services from the Santa Justa train station, coming from Madrid, after being repatriated from Bangladesh.

There he was serving a five-year sentence for drug trafficking.

He is a patient who has had several hospital admissions at UHSM and clinically prosecuted as a paranoid schizophrenic. In prison, the first years he had no antipsychotic medication and recognizes the presence of auditory pseudohallucinations.

**Objectives:**

psychopathological stabilization

**Methods:**

case report

**Results:**

In the psychopathological assessment upon arrival, the patient was hostile and suspicious, even refusing to take food and medication because he was demanding his freedom. He also relates this point to delirious interpretations of passers-by who approached him at the Madrid airport.

During the admission, the patient was referred to Internal Medicine for a global evaluation and analytical tests of his organic situation, finding normocytic anemia without other findings and with good response to the treatment established.

The patient’s psychopathological evolution is very favorable. Progressively more approachable and critical of the phenomena of psychotic nature. Interventions are carried out with Social Work for his overnight stay.

**Conclusions:**

We have the odyssey of one of many patients with a mental illness where their life journey leads them to marginal situations and where elements of a legal nature are intermingled; either by the stay in prison itself or by the need for an admission against their will for psychopathological stabilization and to redirect this shipwrecked life course.

**Disclosure:**

No significant relationships.

